# Assessing and mapping multi-hazard risk susceptibility using a machine learning technique

**DOI:** 10.1038/s41598-020-60191-3

**Published:** 2020-02-21

**Authors:** Hamid Reza Pourghasemi, Narges Kariminejad, Mahdis Amiri, Mohsen Edalat, Mehrdad Zarafshar, Thomas Blaschke, Artemio Cerda

**Affiliations:** 1 Department of Natural Resources and Environmental Engineering, College of Agriculture, Shiraz University, 71441-65186 Shiraz, Iran; 2 Department of Watershed and Arid Zone Management, Gorgan University of Agricultural Sciences and Natural Resources, Gorgan, 49189-434 Iran; 3 Crop Production and Plant Breeding Department, School of Agriculture, Shiraz University, 71441-65186 Shiraz, Iran; 4 Natural Resources Department, Fars Agricultural and Natural Resources Research and Education Center, AREEO, Shiraz, Fars, Iran; 5 Department of Geoinformatics–Z_GIS, University of Salzburg, 5020 Salzburg, Austria; 6 Soil Erosion and Degradation Research Group, Department de Geografia, Universitat de València, València, Spain

**Keywords:** Hydrology, Hydrology, Natural hazards, Natural hazards

## Abstract

The aim of the current study was to suggest a multi-hazard probability assessment in Fars Province, Shiraz City, and its four strategic watersheds. At first, we construct maps depicting the most effective factors on floods (12 factors), forest fires (10 factors), and landslides (10 factors), and used the Boruta algorithm to prioritize the impact of each respective factor on the occurrence of each hazard. Subsequently, flood, landslides, and forest fire susceptibility maps prepared using a Random Forest (RF) model in the R statistical software. Results indicate that 42.83% of the study area are not susceptible to any hazards, while 2.67% of the area is at risk of all three hazards. The results of the multi-hazard map in Shiraz City indicate that 25% of Shiraz city is very susceptible to flooding, while 16% is very susceptible to landslide occurrences. For four strategic watersheds, it is notable that in the Dorodzan Watershed, landslides and floods are the most important hazards; whereas, flood occurrences cover the largest area of the Maharlou Watershed. In contrast, the Tashk-Bakhtegan Watershed is so sensible to floods and landslides, respectively. Finally, in the Ghareaghaj Watershed, forest fire ranks as the strongest hazard, followed by floods. The validation results indicate an AUC of 0.834, 0.939, and 0.943 for the flood, landslide, and forest fire susceptibility maps, respectively. Also, other accuracy measures including, specificity, sensitivity, TSS, CCI, and Gini coefficient confirmed results of the AUC values. These results allow us to forecast the spatial behavior of such multi-hazard events, and researchers and stakeholders alike can apply them to evaluate hazards under various mitigation scenarios.

## Introduction

The Sendai Framework, with its comprehensive vision, recommends more efforts to decrease disaster risk and increase sustainable development. Especially communities who are increasingly susceptible to natural hazards should adhere to these guidelines and plan accordingly. In this regard, the multi-hazard approach is often used in risk reduction projects and studies addressing risks associated with human activities or climate change on a regional and local scale^1^. It is obvious that introducing a universal set of multi-hazard assessment techniques is of fundamental importance for reducing disaster risk, and constitutes a valuable asset to share with other stakeholders, including the private sectors, local governments, and other stakeholders.

The use of the term multi-hazard in the current research is related to the objective of risk reduction among natural hazards, including flood, landslides, and forest fires, in a specified spatial distribution in this study^2,3^. Recently, susceptibility modeling approaches related to single processes have advanced considerably for river floods^4^ and landslides^5–7^. However, there is still neither a common terminology nor a uniform conceptual approach for analyzing multiple hazards in conjunction. This is not unexpected because multi-hazard analyses are not the sum of single-hazard examinations. The various hazard characteristics and the methods used to analyze them are completely different^8^. A variety of quantification measures and susceptibility descriptions exist, which need to be adapted to enable the comparison of multiple hazards^9^. Also, natural processes have various effects on different elements at risk, and the techniques used to determine vulnerability diverge between hazards^3^. These topics constitute the main challenges for multi-hazard analyses.

The possibility of predicting which areas are susceptible to a specific type of disaster, including landslides or forest fires, is undisputed. The prediction techniques have proven valuable for predicting various characteristics of a natural disaster that has occurred^10^. Many researchers recognized that the occurrence of landslides and forest fires is influenced by various aspects that involve human activities and climate conditions^11,12^. Several methods for spatially modelling landslides and forest fires have been developed^13,14^.

Moreover, floods affect more than 20,000 human-lives per year^15^. In Asia, approximately 90% of all human losses are caused by floods^16,17^. A flood that occurred in the center of Fars Province (Shiraz City) on March 25, 2019, killed 21 persons, while injuring 164 others and damaging 1186 homes. In terms of economic impact, financial losses were estimated to be about $ 9,344,615 (http://www.irna.ir/fars/fa/News/83266320). In recent years, with the help of GIS and RS technology, the accuracy of flood susceptibility maps has been improved. Techniques include frequency ratio, logistic regression^18^, weights-of-evidence^19^, fuzzy logic^20^, artificial neural networks^21^, decision tree^22^, support vector machines (SVM)^23^, and Random forest models^24^. In this study, the RF model was selected because it is a very fast machine learning method. It produces an accurate classifier with an internal unbiased estimate of generalizability during the forest building processes^25^. It makes no statistical assumptions, and it is characterized by high prediction performance^13,26^.

In the present study, the assessment was carried out for Fars Province (133,400 km^2^), which is strongly affected by floods, landslides, and forest fires. However, the area is also influenced by other climatic hazards (such as gully erosion), which are not systematically recorded at a municipal level. In this research, the first step was to assess the importance of effective factors on flood, landslide, and forest fire occurrence using the Boruta algorithm. Next, the aim was to prepare susceptibility maps for different hazards using the RF data mining algorithm. Then, the three risks were combined in a multi-hazard probability index (MHPI) with respect to their occurrence probability and the range of susceptibility classes. Based on extensive literature review and to the best of our knowledge, no research related to the multi-hazard modeling of floods, landslides, and forest fires exists to date.

## Study Area

The study area is in the Fars Province (SE Iran) between 27° 2′ to 31° 42′ N latitudes and 50° 42′ to 55° 36′ E longitudes, with an area of approximately 133,400 km^2^ of mainly arid and semi-arid land^27^. It covers 8.1% of Iran and includes 26 cities (Fig. S1). The Fars Province has three different atmospheric regions: First, the mountainous areas in the north and northwest with moderately cold winters and moderate summers. Second, the central regions with rainy winters and hot, dry summers. And, third, the southern and southeastern regions with cold winters and hot summers^28^. The geology of the study area is shown in Table S1.

## Material and Methods

**Figure 1:**
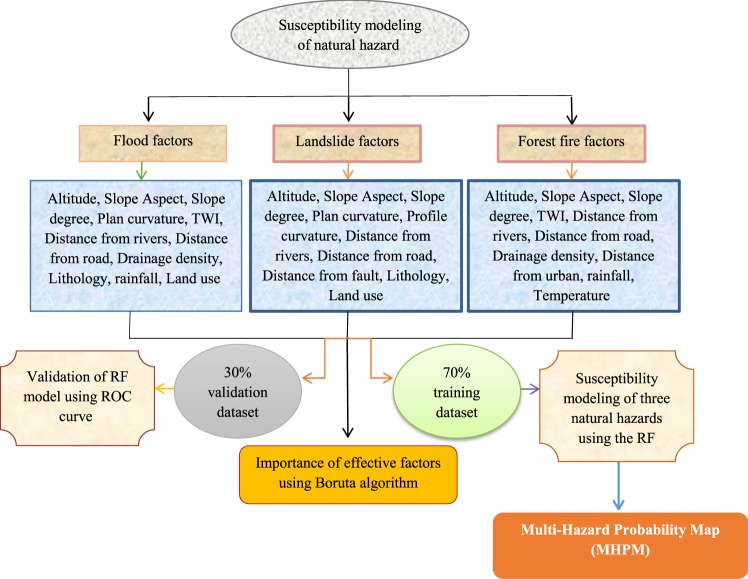
Flowchart of methodology used for multi-hazard spatial modeling in the Fars Province, Iran.

The methodology of the presented study is shown in Fig. 1. The flow chart comprises three main steps, namely 1) data preparation, i.e. obtaining the location of 365 floods, 358 forest fires, and 179 landslides based on intensive fieldwork using a Global Positioning System (GPS) and different province reports; 2) recognizing the most important factors contributing to the occurrence of floods, forest fires, and landslides using the Boruta algorithm; 3) constructing flood, forest fire, and landslide susceptibility maps along with validation processes using the RF model; and, finally, preparing a MHPI in the study area.

### Gathering data related to flood, forest fire, and landslide hazards

Detailed terrain mapping was carried out to locate and recognize floods, forest fires, and landslides. Also, the position of these three hazards was recorded using a GPS receiver. All data were used to verify the locations of these hazards mapped during the fieldwork. The susceptibility modeling technique applied in this study requires samples of both hazards and non-hazards to generate susceptibility maps. Of the total recorded hazard events (365 floods (Fig. S2a), 358 forest fires (Fig. S2b), and 179 landslides (Fig. S2c)) that occurred in the Fars Province, 70% were used in the model building process, while the remaining 30% were used in the validation step (Fig. S2). The same number of non-hazard locations was randomly sampled in the study area. Also, the values of all effective factors were extracted for both samples to validate and train datasets for further processing.

### Construction of flood, forest fire, and landslide conditioning factors

The main factors influencing the occurrence of forest fires are divided into two groups, namely, biophysical and human factors. The biophysical factors are further divided into atmospheric factors (humidity, rainfall, and temperature), and topographic factors (altitude, slope aspect, slope degree). The human factors include land use, access to the forest, and fuel management processes^29,30^. Landslides are influenced by a collection of geo-environmental and anthropological factors^31^. To evaluate the importance of the various effective factors controlling hazard locations, 12 (flood), 10 (forest fire), and 10 (landslide) factors were selected. The factors used in this study to predict the occurrence of flood events are altitude, slope angle, aspect, plan curvature, TWI, distance from rivers, distance from roads, drainage density, lithology, rainfall, land use, and soil features. For landslides, the selected influencing factors are altitude, slope aspect, slope degree, plan curvature, profile curvature, distance from rivers, distance from roads, distance from faults, lithology, and land use. Moreover, for the occurrence of forest fires, the selected effective factors are altitude, slope aspect, slope degree, TWI, distance from rivers, distance from roads, drainage density, distance from urban, rainfall, and annual mean temperature. Topographical factors were extracted using a digital elevation model (ASTER-GDEM) with a spatial resolution of 30 m. Data layers were prepared using ArcGIS 10.2.2 with the pixel size of 30 m^2^. The distance from rivers, roads, and urban maps was obtained from the rivers, roads, and urban areas maps, respectively. The lithology map was obtained from the Geological Survey of Iran at a scale of 1: 100,000. The land use map of the study area was also obtained from the Natural Resources Office of Fars Province at a scale of 1:100,000 and was updated using Google Earth images. Finally, the soil feature map was prepared using data of the soil and water research institute of Fars Province, provided at scale of 1:100,000 and detailed in Table 2S. In general, all effective factor maps are shown in Fig. S3 (a-p).

### Boruta algorithm

The Boruta algorithm was used to prioritize the selected factors affecting natural hazards. The Boruta algorithm is built on the combined dataset by the Random forest classifier and performed in the R statistical package^32^. Boruta is based on a similar viewpoint to that which underlies the Random forest classifier. However, by increasing randomness to the system and collecting results from the ensemble of randomized samples, the devious impact of random variation and relations decrease. Here, this extra randomness shall provide us with a clearer view of which properties are really important^33^. This algorithm has been successfully applied in predicting gully erosion in Iran^34^.

### Random forest data mining model

Random forest (RF) is a supervised classifier^35,36^ that consists of many decision trees and has low error in contrast to other classification algorithms. In this study, the number of trees, minimum node size, and the number of features were used to split each node^35^. However, if one of the predictors has a much stronger effect on the predicting function than the other factors, that predictor is going to be the top splitter in all the trees. Consequently, all trees are going to be similarly constructed and, hence, correlated. Averaging predictions from correlated trees may not decrease the variance significantly^36^.

### Evaluation of susceptibility maps produced by random forest

The area under the ROC curve (AUC) indicates the capability of a model to properly predict the occurrence or non-occurrence of landslides, forest fires, and floods. The ROC curve represents the trade-off between two rates (the false-positive and true-positive rates on the X and Y axes). The AUC values are interpreted as reflecting the following model accuracies: 0.6–0.7 poor, 0.6–0.7 medium, 0.7–0.8 good, 0.8–0.9 very good, and 0.9–1 excellent^37,38^. In the current study, different techniques and measures were applied to evaluate the robustness and uncertainty of the RF model for three different hazards, namely, floods, forest fires, and landslides. These accuracy measures are the true positive rate (TPR), false positive rate (FPR), F-measures, fallout, sensitivity, specificity, true skill statistics (TSS), overall accuracy, corrected classified instances (CCI), and the Gini coefficient^39–41^. All of these indices were calculated based on the four parameters of true negative (TN), false positive (FP), false negative (FN), and true positive (TP).

The TPR (sensitivity) and TNR (specificity) show the probability of correct predictions of the positives and negatives as observed in the reality. The FPR (1– specificity) indicate the probability of incorrect predictions of non-event location as an event. TSS also measure the ability of a predicted value to discriminate between the events and non-events, using all of the elements in the confusion matrix^42^. The CCI considers TN and FN for true- and false-negative predicted events, and TP and FP for true- and false-positive, respectively. The coefficient of variation may often be suggested over the Gini coefficient if a measure of relative precision is selected to evaluate inequality^43^.

1 $$TPR=TP/(TP+FN)$$

2 $$FPR=FP/(TN+FP)$$

3 $${\rm{F}}-{\rm{measure}}=2\,\ast \,{\rm{precision}}\,\ast \,{\rm{recall}}/({\rm{precision}}+{\rm{Recall}})$$

4 $${\rm{Precision}}={\rm{TP}}/({\rm{TP}}+{\rm{FP}})$$

5 $${\rm{Recall}}={\rm{TPR}}$$

6 $${\rm{Fallout}}={\rm{FP}}/({\rm{TP}}+{\rm{FP}})$$

7 $${\rm{Specificity}}={\rm{TN}}/({\rm{TN}}+{\rm{FP}})$$

8 $${\rm{Sensitivity}}={\rm{TP}}/({\rm{TP}}+{\rm{FN}})$$

9 $${\rm{TSS}}={\rm{Sensitivity}}+{\rm{Specificity}}-1$$

10 $${\rm{CCI}}=({\rm{TN}}+{\rm{TP}}/({\rm{TN}}+{\rm{TP}}+{\rm{FP}}+{\rm{FN}}))\ast 100$$

11 $${\rm{Gini}}\,{\rm{coefficient}}=2\ast {\rm{AUC}}-1$$

## Results

### Prioritizing and determining effective factors using the Boruta algorithm

The first aim of using the Boruta algorithm was to select the best conditioning factors for the occurrence of landslides, forest fires, and floods. The resulting rank of features for these three hazards according to their importance is shown in Tables 1–3. According to the mean importance for the occurrence of flood events depicted in Table 1, land use (33.23), drainage density (21.21), and TWI (20.97) are the most important factors, followed by distance from rivers (14.07), aspect (12.39), lithology (12.07), distance from roads (9.81), rainfall (9.70), slope (8.74), plan curvature (7.43), altitude (6.15), and soil (2.99). The highest rank of effective factors for the occurrence of forest fires was assigned to closeness to residential areas (35.36), slope (20.07), aspect (15.03), rainfall (13.48), distance from rivers (9.46), annual mean temperature (8.64), TWI (6.40), and land use (2.71) (Table 2). However, distance from roads (2.24) and altitude (0.08) were found to have no relevance among all considered factors. Additionally, the ranking of effective factors for the occurrence of landslides assigned the highest value to slope (15.95), followed by distance from rivers (12.56), lithology (10.50), land use (8.20), profile curvature (7.08), aspect (6.29), altitude (5.85), and distance from faults (4.82) (Table 3). Distance from roads (1.07) and plan curvature (0.09) were rejected based on this algorithm.

**Table 1:** Considering flood variables importance using by Boruta algorithm.

Factors	Mean Importance	Median Importance	Min Importance	Max Importance	Decision
**Altitude**	6.15	6.08	3.19	8.39	Confirmed
**Aspect**	12.39	12.41	10.53	14.34	Confirmed
**Slope**	8.74	8.74	6.77	10.92	Confirmed
**Plan curvature**	7.43	7.50	5.29	9.14	Confirmed
**Distance from roads**	9.81	9.84	7.63	11.63	Confirmed
**Distance from rivers**	14.07	14.24	11.80	16.46	Confirmed
**Drainage density**	21.21	21.34	19.65	22.90	Confirmed
**Rainfall**	9.70	9.73	7.98	11.68	Confirmed
**TWI**	20.97	20.83	18.17	22.96	Confirmed
**Lithology**	12.07	12.12	10.03	13.55	Confirmed
**Land use**	33.23	33.38	30.74	35.60	Confirmed
**Soil**	2.99	2.92	0.85	4.72	Confirmed

**Table 2:** Considering forest fire variables importance using by Boruta algorithm.

Factors	Mean Importance	Median Importance	Min Importance	Max Importance	Decision
**Distance from rivers**	9.46	9.48	6.66	12.59	Confirmed
**Residential areas**	35.36	35.29	32.15	39.12	Confirmed
**Distance from roads**	2.24	2.26	0.37	4.18	Rejected
**TWI**	6.40	6.46	3.36	8.66	Confirmed
**Slope**	20.07	20.21	17.38	23.16	Confirmed
**Rainfall**	13.48	13.42	10.04	16.94	Confirmed
**Altitude**	0.08	0.18	−1.71	2.12	Rejected
**Aspect**	15.03	15.11	10.96	17.83	Confirmed
**Temperature**	8.64	8.64	6.80	11.19	Confirmed
**Land use**	2.71	2.81	−0.65	4.83	Confirmed

**Table 3:** Considering landslides variables importance using by Boruta algorithm.

Factors	Mean Importance	Median Importance	Min Importance	Max Importance	Decision
**Plan curvature**	0.09	0.03	−2.04	1.73	Rejected
**Aspect**	6.29	6.21	4.43	8.72	Confirmed
**Altitude**	5.85	5.86	2.93	8.84	Confirmed
**Distance from faults**	4.82	4.87	1.67	7.21	Confirmed
**Distance from roads**	1.07	1.17	−1.93	3.23	Rejected
**Distance from rivers**	12.56	12.53	10.31	14.22	Confirmed
**Profile curvature**	7.08	7.12	5.30	9.61	Confirmed
**Slope**	15.95	15.90	13.51	18.58	Confirmed
**Lithology**	10.50	10.47	8.76	12.26	Confirmed
**Land use**	8.20	8.20	6.64	10.90	Confirmed

### Providing natural hazard susceptibility maps using the RF model

**Figure 2:**
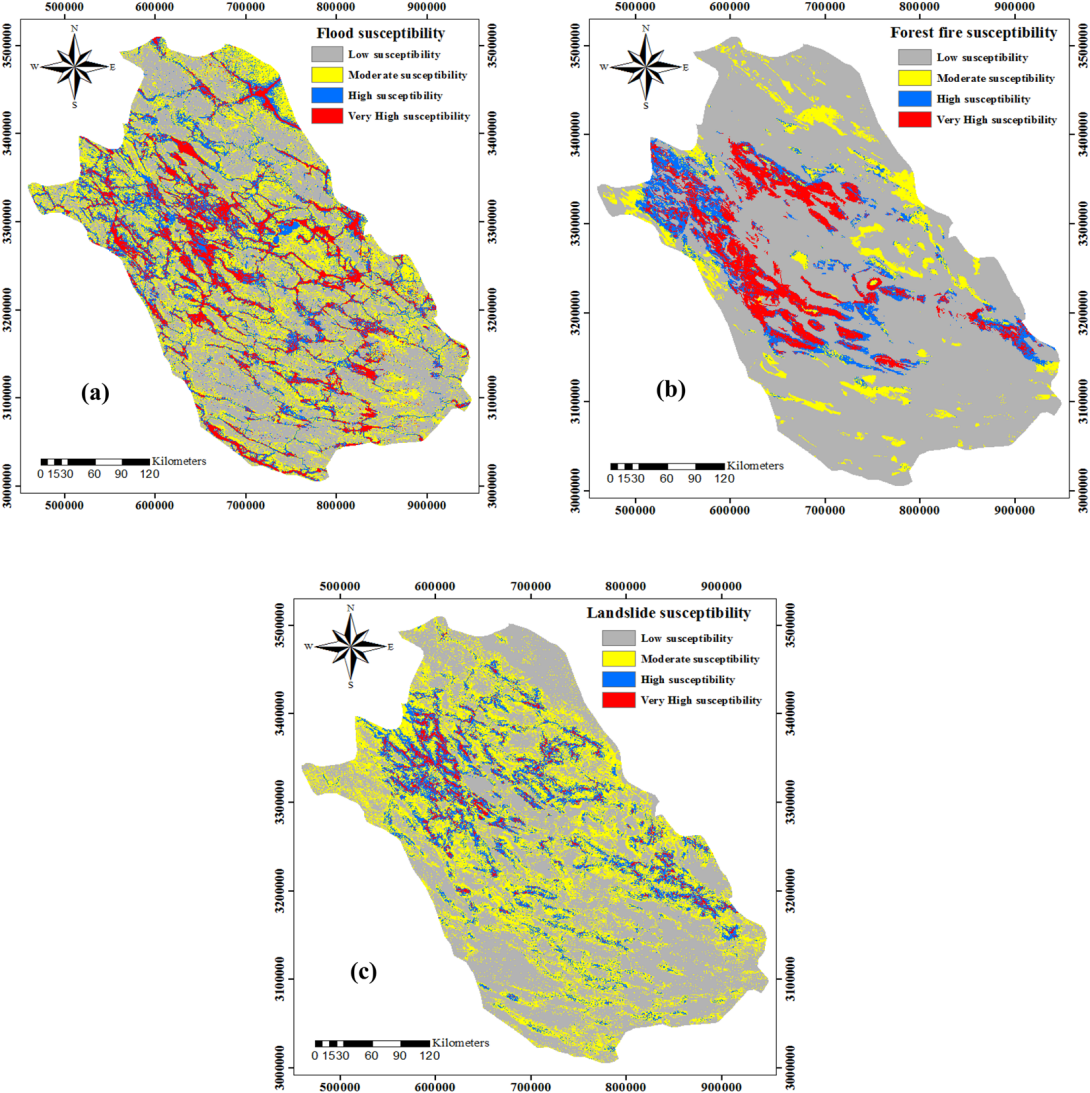
The susceptibility maps of three natural hazards produced using the random forest model.

**Figure 3:**
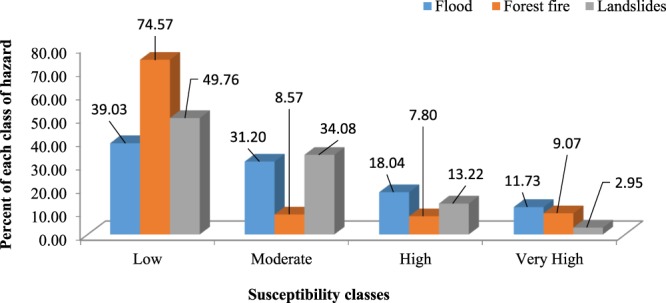
The susceptibility maps of three natural hazards produced using the random forest model.

In this study, susceptibility maps were produced for three natural hazards using the RF model (Fig. 2). Based on the flood susceptibility map produced by the RF model, 39.03%, 31.20%, 18.04%, and 11.73% of the total area in Fars Province are considered as having low, moderate, high, and very high flood susceptibility, respectively (Figs. 2a and 3). According to the forest fire susceptibility map, 74.57%, 8.57%, 7.80%, and 9.07% of the total area was classified into low, moderate, high and very high classes of susceptibility, respectively (Figs. 2b and 3). Also, the landslide susceptibility map derived from the RF model implied that the largest part of the study area (49.76%) has low susceptibility to landslide occurrence, 34.08% of the area has medium susceptibility to landslide occurrence, while the high and very high susceptibility classes cover 13.22% and 2.95% of the area, respectively (Figs. 2c and 3). The spatial aggregation of these three susceptibility maps produced by the RF model confirmed that the majority of the study area has a low susceptibility to the occurrence of flood, forest fire, and landslide events.

**Figure 4:**
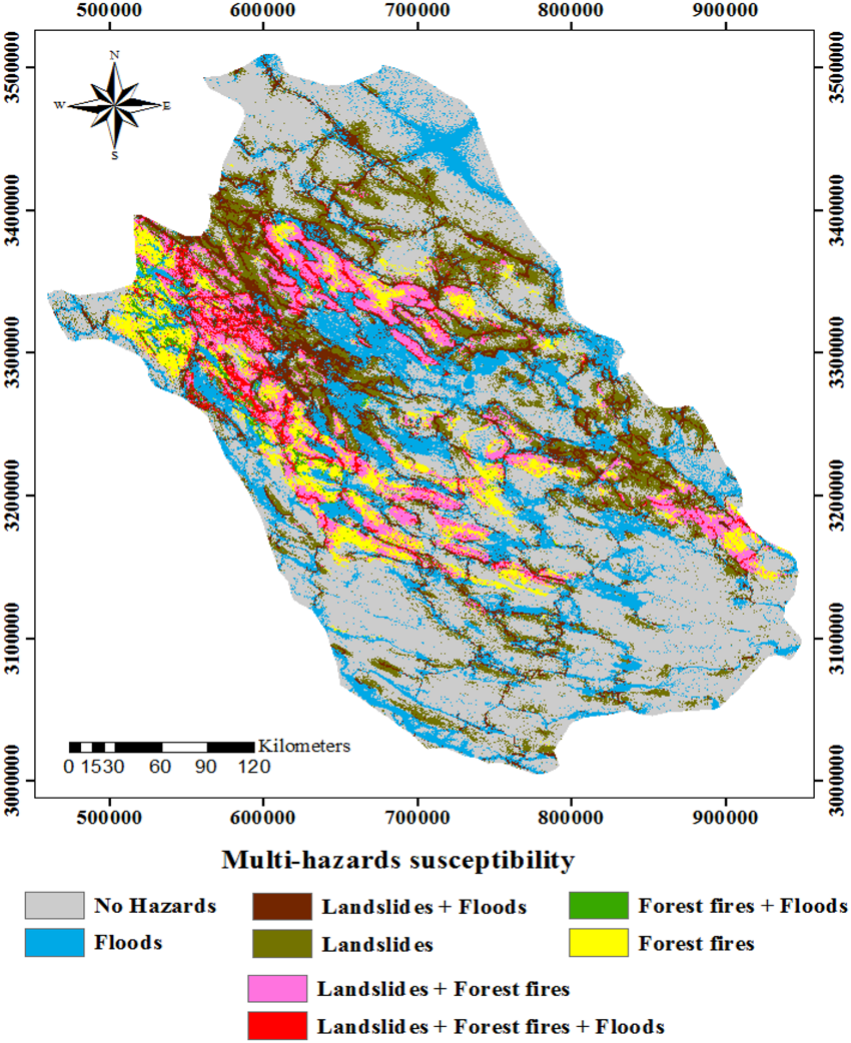
Percentages of susceptibility classes of multi-hazard in Fars province.

In order to produce a multi-hazard probability map (Fig. 4), all three hazard susceptibility maps were considered together. The susceptibility classes of the multi-hazard map produced for Fars Province confirmed that 42.83% of the study area is not susceptible to any hazards. Meanwhile, areas of 17.26%, 5.95%, and 14.16% were at found to be at risk of floods, landslides, and forest fire, separately. Regarding multi-hazard susceptibility, 0.95% of the study area was found to be at risk of floods and forest fires together. Moreover, the combined risk of forest fires and landslides was detected for 7.28% of the study area. Regarding the combined risk of floods and landslides, 8.87% of the study area was deemed susceptible, while, finally, 2.67% of Fars Province was found to be at risk of all three hazards together (Fig. S4).

### Considering natural hazards in Shiraz City (center of Fars Province)

**Figure 5:**
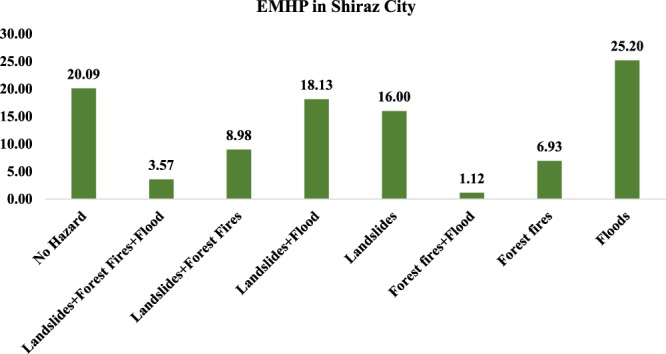
EMHP and percentage of each hazard in Shiraz City.

The results of our multi-hazard analysis (Fig. 5) presented for Shiraz City indicated that 25% of Shiraz City is very susceptible to flood occurrence, whereas about 1.12% of the study area is susceptible to both floods and forest fires. Furthermore, 18.13% and 3.57% of Shiraz City face the combined risk of landslides and floods, respectively landslides, forest fires, and floods. The flood event that occurred on March 25, 2019, which killed 21 persons, injured 164 others, damaged 1,186 homes, and caused financial losses around $ 9,344,615, impacted two areas, in particular, namely the Quran Gate and Saadi Zone (http://www.irna.ir/fars/fa/News/83266320). One of the most important findings and achievements of this study is that the prepared EMHM could very accurately predict flood events in the areas of Quran Gate and Saadi Zone.

### Studying the susceptible watersheds of natural hazards

**Figure 6:**
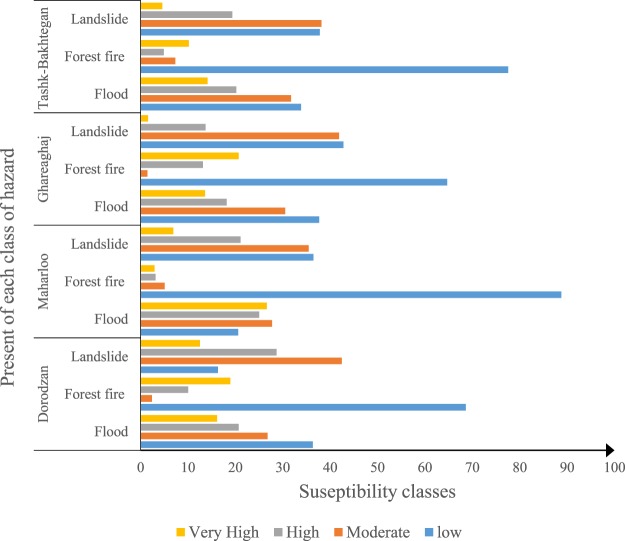
Percentages of susceptibility classes of multi-hazard in Dorodzan, Maharlou, Ghareaghaj, and Tashk-Bakhtegan Watershed.

Dorodzan Watershed is one of the strategic areas of Fars Province and plays a very important role in the agricultural production and self-sufficiency of Iran. It is an area which supplies water resources to Tashk and Bakhtegan lakes and is affected by wind erosion. Figure 6 illustrates that 36.35% and 68.64% of Dorodzan Watershed are covered by the low class of susceptibility to flood and forest fire, respectively. However, regarding landslides, the moderate susceptibility class covers the largest area (42.45%). The Maharloo Watershed, as a second grade watershed of the Ministry of Energy, is the main source of the Kor River. In this watershed (Fig. 6), the moderate class covers the largest area (27.76%), although the classes of low susceptibility to forest fires (88.81%) and landslides (36.48%) covered the greatest area in Maharloo Watershed. The Ghareaqaj Watershed, which is currently used for drinking and agricultural purposes, is one of the most important rivers in Fars Province. The construction of the Salman Farsi Dam in Qir and Karzin and studies on the construction of the Kavar Dam on this river indicates the importance of the river in the mentioned province. In this watershed (Fig. 6), all three hazards (floods, forest fires, and landslides) pose a low risk (37.68%, 64.71%, and 42.80%). Moreover, the most important source of water supply are the Bakhtegan and Tishak lakes. Based on Fig. 6 (Tashk-Bakhtegan watershed), the low susceptibility class covers the largest area of flood (33.85%) and forest fire (77.59%), while, based on the landslide susceptibility map produced by the RF model, 38.19% of the total area was covered by the moderate class.

### Studying the validation of natural hazard susceptibility maps

In order to produce natural hazard susceptibility maps, all hazards were divided into two data sets: one for modeling and one for validation. The accuracy of the three maps produced by the RF model was verified using ROC curves (Table S3). The AUC values for the flood, forest fire, and landslide maps were 0.834, 0.943 and 0.939, respectively. Regarding the standard error, floods had the highest value (0. 028), followed by forest fires (0.016), and landslides (0.023). Further, the forest fire map had excellent accuracy (0.958), while the model considered the landslide and flood maps as very good. Also, the results of the other measures (Table 4) confirmed the accuracy of the three hazard maps, as, according to Table 4, the F-measure, specificity, and sensitivity of each hazard is more than 0.77. Furthermore, the TSS index is 0.541 for floods, which indicates fair accuracy, whereas its values for landslides and forest fires were 0.889 and 0.850, respectively, indicating an excellent model, based on the findings of Allouche *et al*. (2006)^44^. Also, according to published reports, a Gini coefficient value above 0.6 (60%) indicates a good model in terms of accuracy. On the other hand, when the CCI (overall accuracy) is between 0.6–0.8, it shows that the accuracy of the model is good. So, the RF model is known as an accurate classifier for the three depicted hazards.

**Table 4:** Different robustness measures for validation of the built model of each hazard.

Hazards	TN	FP	FN	TP	TPR	FPR	F-measures	Fallout	Specificity	Sensitivity	TSS	CCI	Gini
**Flood**	84	25	25	84	0.771	0.229	0.771	0.229	0.771	0.771	0.541	77.06	0.668
**Landslides**	48	6	6	48	0.889	0.111	0.889	0.111	0.889	0.889	0.778	88.89	0.878
**Forest fire**	91	16	16	91	0.850	0.150	0.850	0.150	0.850	0.850	0.701	85.05	0.886

## Discussion

In this study, the importance of factors controlling landslide, flood, and forest fire locations was analyzed using the Boruta algorithm. The Boruta algorithm provided quantitative results, which is a significant advantage that allows the potential comparison of studies in different regions around the world. As it was already stated, the study area is prone to combinations of landslides, floods, and forest fires. Generally, the development and formation of these natural hazards are controlled by several factors, and the distribution of these hazards cannot be random.

The most statistically significant relationship among factors, based on the Boruta algorithm, was found between flood location and land use, and land use presented as the most important factor influencing flood hazards among all considered variables. Wheater and Evans (2009)^45^ implied that land use affects the hydrology that determines water resources leading to flood hazards. It is increasingly identified that the management of water and land are strongly linked. Generally, steeper slopes are more vulnerable to massive erosion, including landslides. The steepness of slopes is reported as a factor of primary importance that promotes high runoff velocity, which results in this type of erosion. Regarding slope and aspect, forest fires predominantly occur in the steep slopes of the southern areas, as vegetation is typically dry. The effects of slope and aspect on fire behavior in the occurrence of forest fires have been reported by Adab *et al*. (2013)^46^. According to Pourghasemi (2016)^47^, topographic data (i.e. slope and aspect) are the most important factors for forest fire assessment. Contrary to the above results, Bui *et al*. (2017)^48^ found that NDVI (Normalized Difference Vegetation Index) had the strongest impact on the occurrence of forest fires. However, Hong *et al*. (2017)^49^ and Gigovic *et al*. (2019)^50^ respectively demonstrated that slope has a significant positive effect on the occurrence of forest fire events. In relation to flood effective factors, the research carried out by Liu *et al*. (2005)^51^ confirmed that the urbanization scenario has a strong influence on heightening flood volume. For instance, afforestation has a positive impact, while deforestation has a negative impact on the occurrence of floods.

Besides determining variable importance, the RF model was used to prepare susceptibility maps for landslides, floods, and forest fires, first separately (Fig. 2), and then jointly in the form of a multi-hazard map (Fig. 4). The susceptibility maps for floods, landslides, and forest fires revealed that most of the study area is characterized by low susceptibility to each hazard when analyzed separately (Fig. 3). The multi-hazard probability map modeled by RF revealed that the most parts of the study area are not susceptible to any hazards, whereas few areas are at risk of all three hazards together (Fig. 3). Floods are recognized as the most dangerous hazard in the study area, followed by landslides and forest fires (Fig. S4). Further, effective flood risk reduction requires more analysis of this individual hazard and its interaction with the other hazards. Additionally, the validation of the RF models determined an excellent accuracy of the forest fire and landslide susceptibility maps (Table S3). Pourghasemi *et al*. (2019)^52^ produced a susceptibility map for three hazards (i.e. landslides, floods, and earthquakes) using the ensemble model named SWARA-ANFIS-GWO. They showed that 17.14% of the area is affected by no hazards, whereas most parts were susceptible to landslide and flood hazards together (33.70%). They also indicated accuracies of 84% and 80% for flood and landslide maps, respectively. Skilodimou *et al*. (2019)^53^ applied the analytical hierarchy process (AHP) to produce separate maps for landslide, flood, and earthquake hazards and combined them into a single multi-hazard map. They showed that 80% of the landslide occurrences and all the recorded flood events fall within the boundaries of the moderate, low and very low susceptibility classes.

There are several advantages that make the RF model suitable for the approach in the present study. First, it is a simple, fast algorithm that makes no statistical assumptions and is characterized by a high prediction performance^54,55^. It produces an internally unbiased evaluation of generalizability with an accurate classifier during the forest building processes^26^ and provides better consistency of results and robustness of forecasts^56^. The RF can precisely handle heterogeneous inputs of different nature and scalability from different sources^55,57^. Another important benefit of the RF model is that there are significant criteria that indicate the importance of each predictor variable^55,58^. However, it has some sources of uncertainty that are frequently unacknowledged or even unrecognized.

One source of uncertainty in the modeling process is related to the gathered data. It is important to consider non-linear correlations among dependent and independent variables; this problem can be solved by machine learning techniques. One of the advantages of machine learning techniques in comparison to traditional methods (bivariate and multivariate statistical methods) is that the ML algorithms can deal with noises in the data and are also accurate in the presence of uncertain data and limited measurement errors. Quality of data is also important. In the current study, different extensive field surveys were conducted to collect suitable data for all three hazards; however, according to the accuracy of the flood susceptibility map (Tables S3 and 4), there appears to be greater uncertainty compared to the landslide and forest fire hazards, because the selection of flood locations is so difficult compared to other hazards. Another uncertainty source is the accuracy of the built model. For solving this problem, different techniques were applied, and the results are presented in Table 4. According to Table 4, the achieved results of the AUC values confirmed the accuracy of the built model for the three examined hazards, namely floods, landslides, and forest fires. Also, dividing the entire dataset into two sets for training (70%) and validation (30%) can be effective in decreasing uncertainties in a model’s performance. Another uncertainty source is limitations of the learned model that the ML techniques such as the RF isn’t faced to this problem, meanwhile this algorithm for removing this uncertainty, used from error rates (Table 4) and out-of-bag indicator. Results of the out-of-bag values for forest fires, landslides, and floods were 3.55%, 15.6%, and 22.27%, respectively.

Nowadays, the necessity of using machine learning techniques is increasingly emphasized in the susceptibility modeling of geomorphological features and processes^37^. A universal framework describing which factors to compare is required. This general framework can be semi-quantitative, qualitative, or quantitative^3^. It should be suitable for both single hazard and multi-hazard assessments, because multi-hazard evaluation plays the main role in reducing disaster risk and provides crucial information for sharing with the other stakeholders, such as local governments and private sectors^55^. Considering multi hazards jointly and applying the same technique to analyze them can give us a comprehensive view of the changes occurring in the environment. Further, a synthesized multi-hazard probability map supports planners in sustainable development and adaptive management because this map provides homogenized information about different environmental hazards for a specific area^64^. It means that the potential use of hazard evaluation becomes obvious when considering all hazards together, on the basis of which plans and projects can be implemented considering this comprehensive view of a region^59^. From this point of view, a multi-hazard probability map can be used for integrated and comprehensive watershed management and land use planning and, consequently, for the sustainable development of a region.

## Conclusion

A better understanding of the factors controlling flood, forest fire, and landslide occurrence is crucial to the sustainable development of regions prone to these three hazards, such as the Fars Province. In this study, 365 floods, 358 forest fires, and 179 landslides were mapped for an area of 133,400 km^2^. The Boruta algorithm enabled us to analyze the impact of effective factors on the occurrence of three different natural hazards. According to the Boruta algorithm, the most important factor controlling flood occurrence in the study area was land use, followed by drainage density, and TWI. Among the different factors controlling forest fire occurrence, residential areas ranked highest, followed by slope, and aspect. Moreover, the highest rank of conditioning factors regarding landslide occurrence was found to be slope, followed by distance from rivers, and lithology. The RF model was also applied to prepare a susceptibility map of flood, landslide, and forest fire locations. The multi-hazard probability map produced for floods, forest fires, and landslides in Fars Province revealed that the majority of the land is not prone to any hazards. Total areas of 17.26%, 5.95%, and 14.16% were found to be at risk of floods, landslides, and forest fire, separately. However, 2.67% of Fars Province was found to be at risk of all three hazards together. Based on the AUC values, the best accuracy was determined for the forest fire susceptibility map, followed by the maps produced for landslides, and floods. Further, the multi-hazard probability map prepared in this study can be used for integrated and comprehensive watershed management and land use planning and, consequently, for sustainable development in the study region.

## Supplementary information

Supplementary Information.
