# Supplementary material for: Assessing and mapping multi-hazard risk susceptibility using a machine learning technique
Source: Sci Rep. 2020 Feb 21;10:3203. doi: 10.1038/s41598-020-60191-3 (PMC7035287; doi:10.1038/s41598-020-60191-3)
Supplement: Supplementary file 1 — Supplementary Information. [file 41598_2020_60191_MOESM1_ESM.pdf]

Assessing and mapping multi-hazard risk susceptibility using a machine learning technique

Hamid Reza Pourghasemi[1*], Narges Kariminejad[2], Mahdis Amiri[2], Mohsen Edalat[3], Mehrdad Zarafshar[4], Thomas Blaschke[5], Artemio Cerda[6]

[1] Department of Natural Resources and Environmental Engineering, College of Agriculture, Shiraz University, 71441-65186, Shiraz, Iran. Email: hamidreza.pourghasemi@yahoo.com

[2] Department of Watershed and Arid Zone Management, Gorgan University of Agricultural Sciences and Natural Resources, Gorgan 49189-434, Iran. Email: narges.karimi991@gmail.com; mahdisamiri94@gmail.com

[3] Crop Production and Plant Breeding Department, School of Agriculture, Shiraz University, 71441-65186, Shiraz, Iran. Email: edalat@shirazu.ac.ir

[4] Natural Resources Department, Fars Agricultural and Natural Resources Research and Education Center, AREEO, Shiraz, Fars, Iran. Email: mehrdadzarafshar@gmail.com

[5] Department of Geoinformatics–Z_GIS, University of Salzburg, 5020 Salzburg, Austria, thomas.blaschke@sbg.ac.at

[6] Soil Erosion and Degradation Research Group, Department de Geografia, Universitat de València, Spain; artemio.cerda@uv.es

*Email: hr.pourghasemi@shirazu.ac.ir (Corresponding authors)

Table of content

Figure S1. Location of the study area in Iran

Figure S2. Training and validation points of three hazards

Figure S3. Maps of effective factors used for in order to danger of natural hazards in the study area

Figure S4. Percentages of susceptibility classes of three natural hazards

Table S1 Geology of the study area

Table S2 Soil taxonomy of the studied area

Table S3 Predictive performance of the Random forest model in the validation process

Figure S1 Location of the study area in Iran

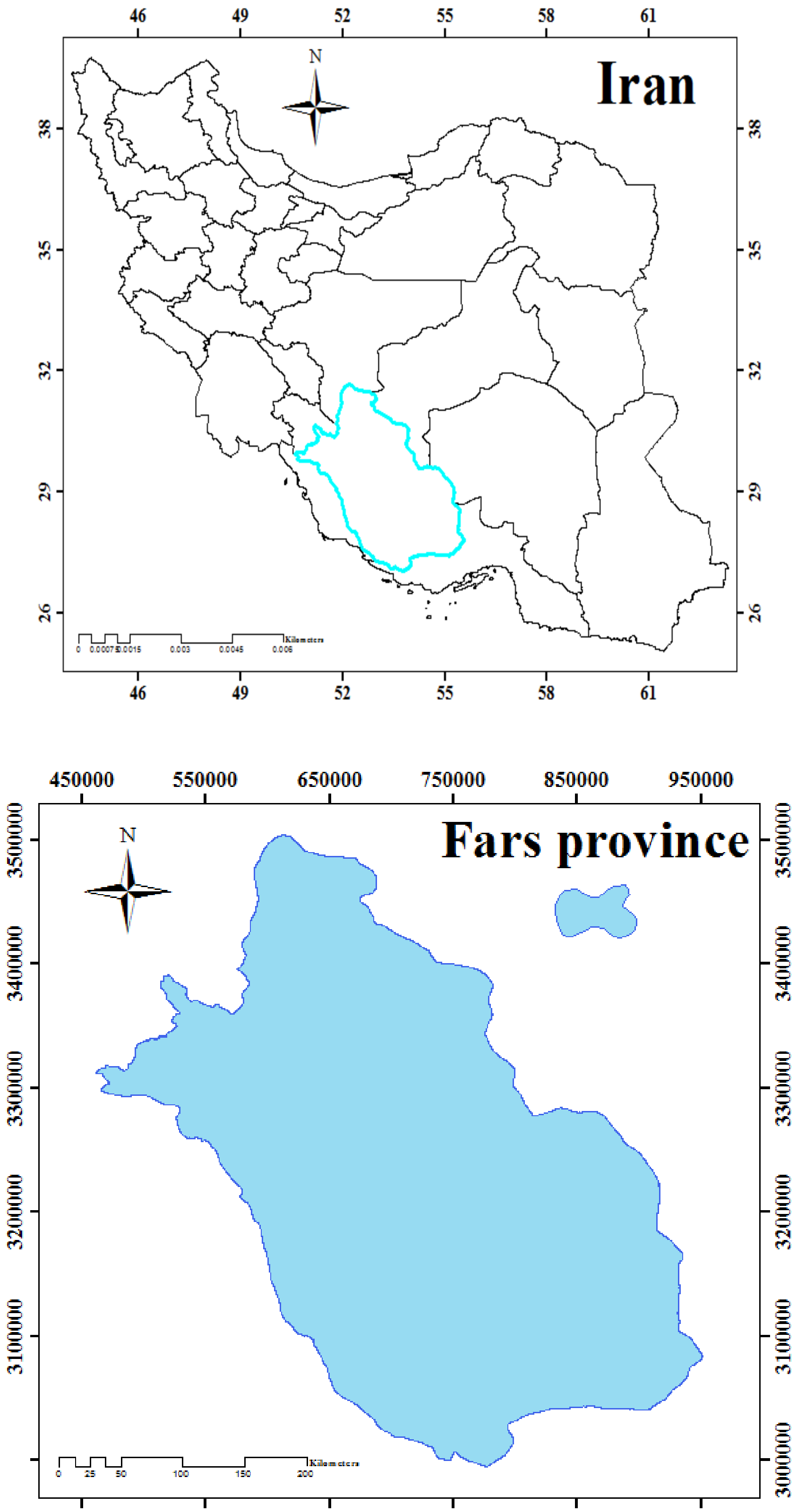

Figure S2 Training and validation points of three hazards

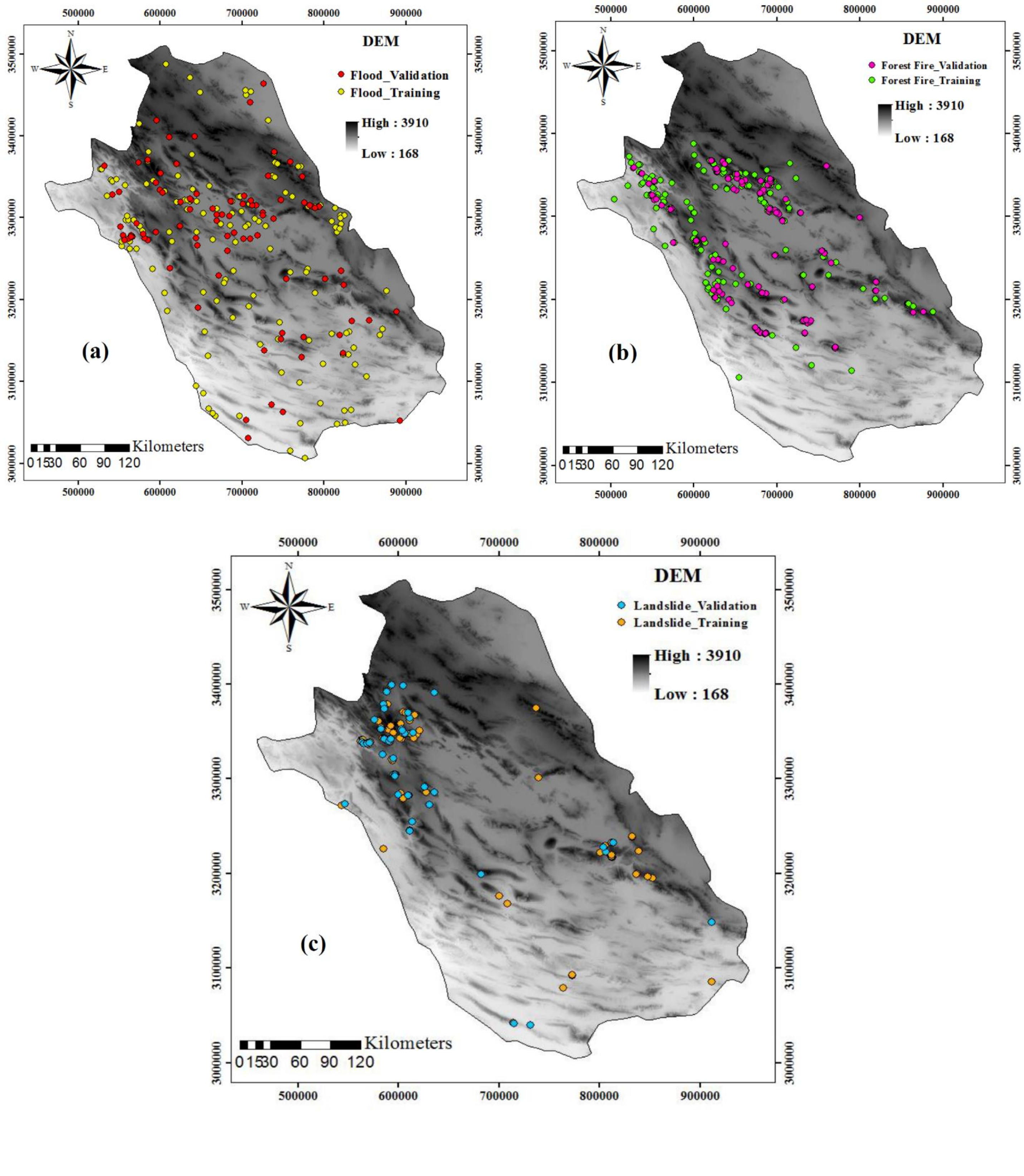

Figure S3 Maps of effective factors used for in order to danger of natural hazards in the study area

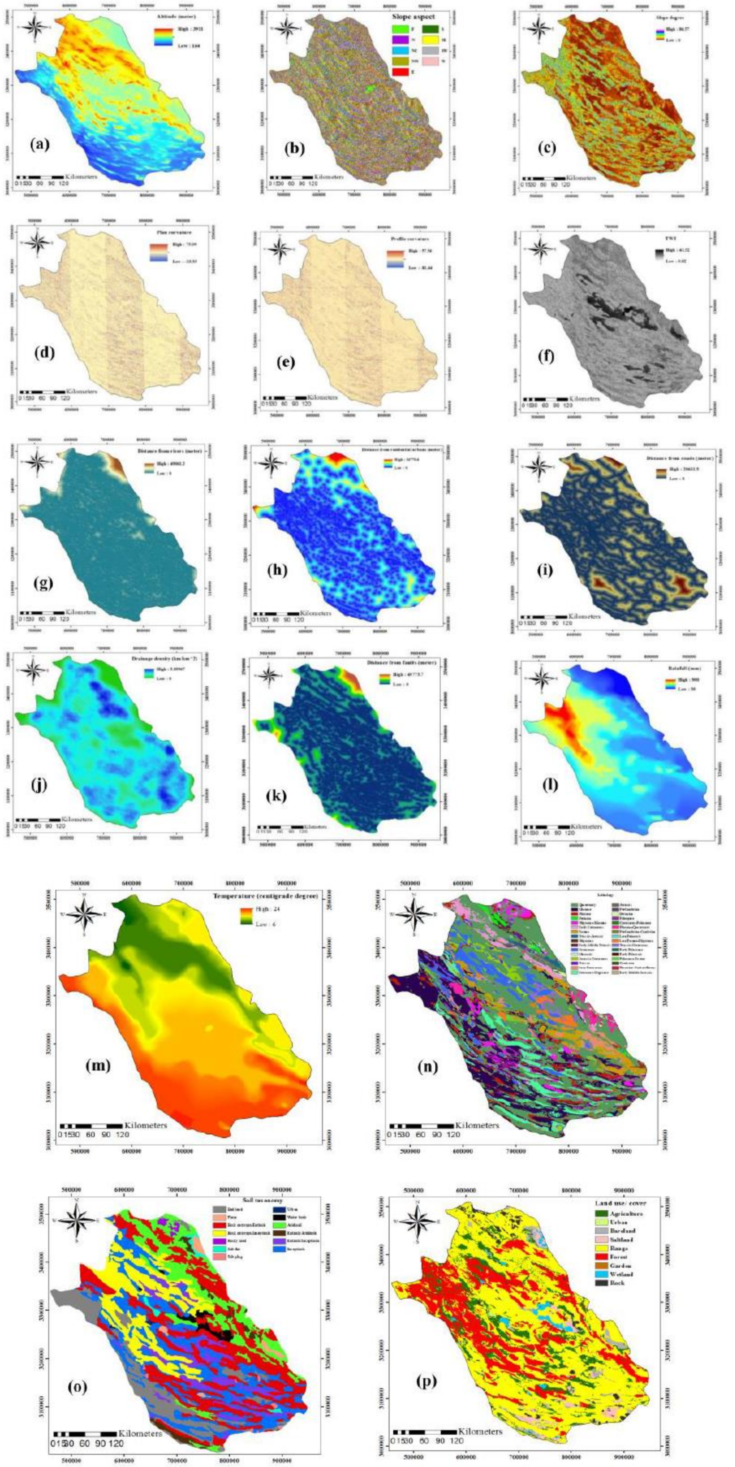

Figure S4 Percentages of susceptibility classes of three natural hazards

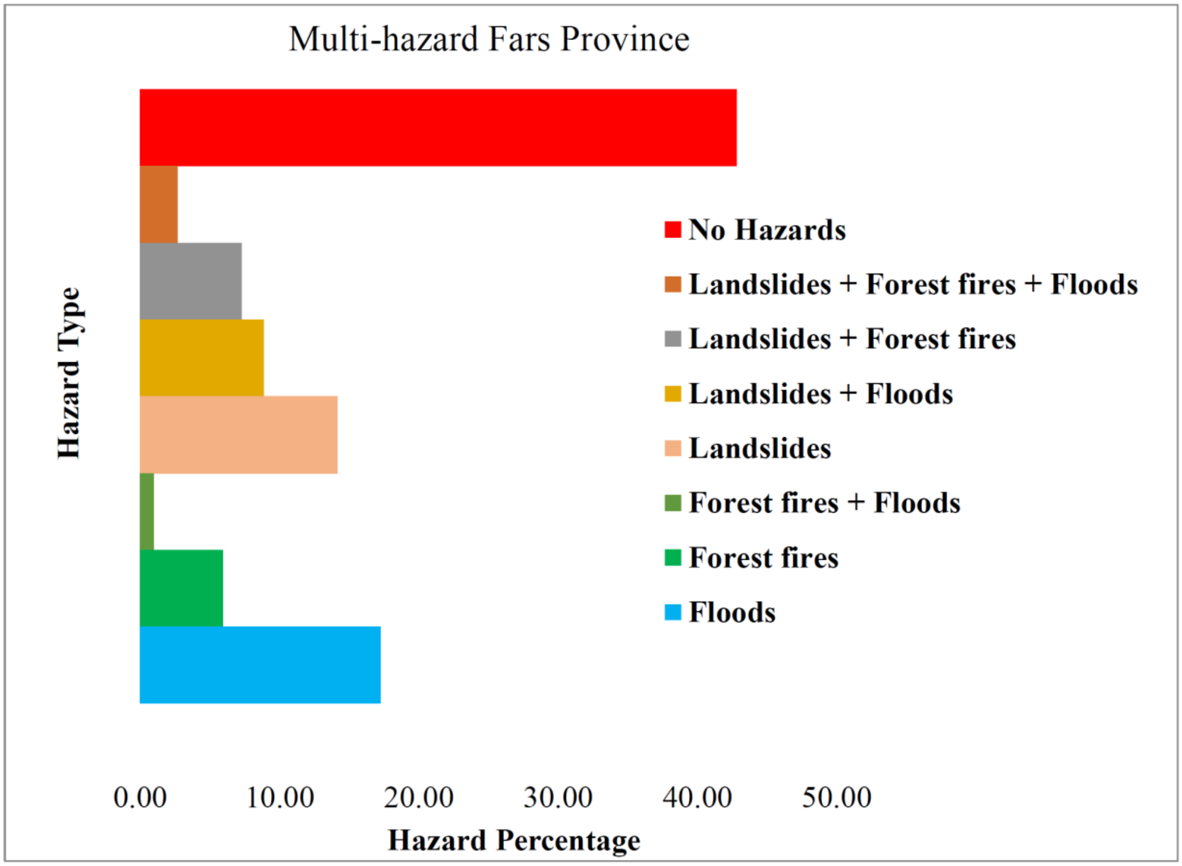

Table S1. Geology of the study area

Formation	Description	Lithological units	Age
**-**	Low level pediment fan and valley terrace deposits	Qft2, Qcf	Quaternary
**Aghajari, Mishan**	Brown to grey, calcareous, feature-forming sandstone and low weathering, gypsum- veined, red marl and siltstone	Mur, MuPlaj,<br>Mmn, MPlfgp	Miocene
**Bakhtyari**	Alternating hard of consolidated, massive, feature forming conglomerate and low -weathering cross - bedded sandstone	Plc, Plbk	Pliocene
**Jamal, Dorud**	Massive to thick - bedded, dark - grey, partly reef type limestone and a thick yellow dolomite band in the upper part	Pj, P	Permian
**-**	Marl with intercalations of limestone	OMqm, OMql	Oligocene-Miocene
**Taft**	Thin to medium bedded argillaceous limestone and thick bedded to massive, grey orbitolina bearing limestone	Klsm, Klsol, Klsm, Ktl	Early Cretaceous
**Jahrum**	Grey and brown weathered, massive dolomite, low weathered thin to medium -bedded dolomite and massive, feature forming, buff dolomitic limestone	Eja, Ek	Eocene
**Shemshak**	Dark grey shale and sandstone	TRJs	Triassic-Jurassic
**Lower Red**	Red and green silty, gypsiferous marl, sandstone and gypsum	Olm,s,c	Oligocene
**Shotori**	Well - bedded, dense, yellow dolomite	TRsh	Early-Middle Triassic
**Gurpi**	Bluish grey marl and shale with subordinate thin - bedded argillaceous -limestone	Kgu	Cretaceous
**Naiband**	Sandstone, quartz arenite, shale and fossiliferous limestone	TRn	Mesozoic
**-**	Undivided Khami Group, consist of massive thin - bedded limestone comprising the following formations : Surmeh,Hith Anhydrite, Fahlian, Gadvan and Dariyan	JKkgp,<br>KEpd-gu	Jurassic-Cretaceous

**Khamehkat and and Neyriz**	Thin to medium - bedded, dark grey dolomite ; thin - bedded dolomite, greenish shale and thin - bedded argillaceous limestone	TRe1, TRkk-nz	Triassic
**Tarbur**	Massive, shelly, cliff - forming partly anhydritic limestone	Ktb	Late Cretaceous
**-**	Undivided Asmari and Jahrum Formation, regardless to the disconformity separates them	EOas-ja	Paleocene-Oligocene
**Baghamshah**	Pale - green silty shale and sandstone	Jbg, Jf	Jurassic
**Amphibolite Facies**	Medium-grade, regional metamorphic rocks	pCmt1, pCmt2	Pre Cambrian
**Shidhtu**	Alternation of shale, marl and fossiliferous limestone, loccaly with intercalations of quartz arenite	Dsh	Devonian
**Pabdeh**	Blue and purple shale and marl interbedded with the argillaceous limestone	PeEpd	Paleogene
**Amiran**	Dark olive - brown, low weathered siltstone and sandstone with local development of chert conglomerates and shelly limestone	KPeam	Cretaceous-Paleocene
**-**	Fluvial conglomerate, Piedmont conglomerate and sandstone	PlQc	Pliocene-Quaternary
**-**	Rock salt, gypsum & blocks of contorted masses of sedimentary material such as black laminated fetid limestone, brown cherty dolomite, red sandstone & varigated shale in association with igneous rocks such as diabase, basalt, rhyolite and trachyte	pC-Ch	Pre Cambrian-Cambrian
**-**	Granite	PZ2gr	Late Paleozoic
**-**	-	Lake	Late Eocene-Oligocene
**Kerman and Neyzar Radiolarites**	Purple and red thin - bedded radiolarian chert with intercalations of neritic and pelagic limestone	TRKurl, pd	Triassic-Cretaceous
**Barreh Koshan Complex and Rutchan Complex**	Gneiss, anatectic granite, amphibolite, kyanite, staurolite schist, quartzite and minor marble	Pz1mt	Early Paleocene
**-**	Gneiss and anatectic granite	Pz1gn	Early Paleozoic

**Sachun**	Pale red marl, marlstone, limestone, gypsum and dolomite	PeEsa	Paleocene-Eocene
**Lalun**	Dark red meddium - grained arkosic to subarkosic sandstone and micaseous siltstone	Cl	Cambrian
**Sargaz Complex**	Mica schist, green schist, graphite schist, black pyyllit and minor marble	DC2met	Devonian-Carboniferous
**Surmeh**	Thick - bedded to massive dolomitic limestone, thin - bedded argillaceous limestone and marl	Jsm	Early Middle<br><br>Jurassic

Table S2. Soil taxonomy of the studied area

Soil Taxonomy	Description
**Bad land**	Sundry lands when soil erosion increases its depth and width, becomes a bad land, that is one of the nine physiographic units
**Playa**	The shallow lakes located in arid and semi-arid soils, where they are clay and silt, is a barrier to water penetration. It can either be water or not, and if it contains water, it is caused by precipitation and the water is salty so that it salt is extracted
**Rock outcrops/Entisols**	The outcrop is destroyed due to the erosion of all existing materials or soil on the rock and the rock has been exposed./ Immersive soils that are just beginning to evolve, most of them, like the C horizon and bedrock, can also have a A horizon. but the rock outcrops properties are dominant in them
**Rock outcrops/Inceptisols**	The outcrop is destroyed due to the erosion of all existing materials or soil on the rock and the rock has been exposed./ Young soils are the most abundant soils in the world, the soils that begin to form the horizon and have a cosmic horizon, but t the rock outcrops properties are dominant in them
**Rocky land**	Rocky lands usually contain rocks can be angled or non-angular. If the rocks are displaced by water, they are circular and without angle, but if they are not caused by a force, for example, due to the force of gravity and on the basis of weight, they will flow from the mountain. Angle Had been
**Salt flat**	Salt shells are formed in flat lands
**Salt plug**	Holes or hills or salt domes
**Urban**	Urban land affected by human construction
**Water body**	There is a water horizon and the conditions are such that it encloses the water in the area

**Aridisoil**	The soils are arid and semi-arid regions and have a calcic horizon, natric, and gypsic horizons
**Entisols/Aridisols**	Soils that have both Aridisols and Entisols properties, but Entisols properties (immature soils) dominant
**Entisols/ Entisols**	The soil has Entisols region and Entisols propertise, but Entisols propertises is more dominant in the region
**Inceptisols**	Young soils are the most abundant soils in the world, the soils that begin to form the B horizon and have a Cambic horizon

Table S3. Predictive performance of the Random forest model in the validation process

RF model	Area	Standard error	Asymptotic significant	Asymptotic 95% Confidence Interval	
				Lower bound	Upper bound
**Flood**	0.834	.028	.000	.780	0.888
**Forest fire**	0.943	.016	.000	.912	0.974
**Landslide**	0.939	.023	.000	.893	.985
